# Evaluation of fetal heart serum amyloid a concentrations in infectious cattle abortion cases

**DOI:** 10.1016/j.heliyon.2022.e11330

**Published:** 2022-10-31

**Authors:** Zeki Aras, Orhan Yavuz

**Affiliations:** a Department of Microbiology, Aksaray University, Campus, 68100 Aksaray, Turkey; b Department of Pathology, Aksaray University, Campus, 68100 Aksaray, Turkey

**Keywords:** Serum amyloid A, Cattle, Abortion, Fetus, ELISA

## Abstract

Serum amyloid A (SAA) concentrations have been investigated in diseases of human and domestic animals and increased SAA levels have been reported in infectious diseases. In the present study, we determined the fetal heart blood SAA concentrations in aborted bovine fetuses and investigated the relationship between the level of SAA and causative infectious agents. A total of 46 heart blood samples were collected from aborted bovine fetuses between July 2018 and July 2019 and were assigned to two groups according to microbiological, pathological and molecular results. Group 1: An infectious disease was diagnosed by detecting a microorganism (21 cases); Group 2: An infectious or inflammatory disease was not detected (25 cases). The fetal heart blood SAA concentrations were measured by commercial ELISA test. Serum amyloid A concentrations in aborted bovine fetuses were elevated from 6.1 to ≥40 mg/L in 17 of 21 cases in group 1. In group 2, SAA concentrations were less than 2.5 mg/L in 23 of 25 cases. This difference was statistically significant between group 1 and group 2. These findings suggest that SAA concentrations in fetal heart blood from bovine fetuses is potentially a novel marker for distinguishing between infectious and non-infectious bovine abortion cases.

## 1 Introduction

The many bacterial, viral, fungal, and parasitic infections of cattle have adverse effects on cow health involving fertility [1]. The infections can lead to abortion, placentitis, early embryonic death, retained placenta, delay in conception, repeat breeding, anoestrus, and delayed oestrus. These abnormalities will ultimately lead to reduction in calving on dairy farms. The economic losses due to abortion or repeat breeding cases is estimated around 500–900 US$ per case and 4.5–6.7 US$ per day [2, 3, 4]. Fetal malformations and abortions are easy to detect although causes of abortion require detailed work [1].

Serum amyloid A (SAA) is an acute phase protein synthesized predominantly by the liver and regulated by immune system modulators such as interleukin-1 and interleukin-6. The known functions of SAA are immunomodulation, cell migration, cell differentiation, cell proliferation, and invasion [5, 6, 7]. It has been described that SAA is also synthesized by extrahepatic sources, including first-trimester trophoblasts [8]. SAA has immunoregulatory effects and a key role in trophoblastic migration and invasion in these events [9]. SAA regulates trophoblast metalloprotease activity and invasion in the placenta at low concentrations. However, these events are markedly disturbed in the presence of high concentrations of SAA [7]. Serum amyloid A is activated by infection, trauma, inflammation, stress, and toxins [5, 6, 7, 8, 9, 10]. SAA levels usually rise from 6 h to 3 days post-infection [11].

Serum amyloid A concentrations have been investigated in diseases of domestic animals. Increased SAA levels have been associated with neonatal infection, bovine respiratory disease, brucellosis, septicemia, and arthritis. The results of these studies indicated that SAA concentrations could be used as a biomarker for infectious diseases [10, 12, 13, 14, 15].

In a recent study, the fetal heart serum amyloid A concentrations have been investigated in aborted equine fetuses and elevated SAA levels have been reported in infectious abortion cases [10]. However, SAA concentrations in fetal heart blood have not been reported in bovine abortion cases. The aim of this study was to evaluate fetal heart SAA concentrations in aborted bovine fetuses and investigate an association with infection.

## 2 Materials and methods

### 2.1 Materials

A total of 46 late to full-term aborted bovine fetuses, submitted to the Aksaray University Veterinary Diagnostic Laboratory between July 2018 and July 2019 were included in the study. Necropsy of fetuses was performed according to a routine laboratory protocol for investigating the cause of abortion. Tissue fragments from the aborted fetuses, including lung, liver, brain, kidney, spleen and heart, were taken and fixed in 10% formalin solution. Following fixation, tissue samples were subjected to routine processing and stained with Hematoxyline-Eosine for histopathological examination [16]. Samples of lung, liver, brain, kidney, spleen, heart and abomasum contents were used for the detection of the major infectious agents of abortion, namely *Brucella* spp., *Campylobacter* spp., *Salmonella* spp., *Listeria* spp., *Chlamydophila abortus*, *Coxiella burnetii*, Bovine viral diarrhea virus, Bovine herpes virus-1, fungi and yeasts. The selective mediums were used for isolation of *Brucella* spp., *Campylobacter* spp., *Salmonella* spp. and *Listeria* spp. PCR methods were carried out for detection and identification of *Brucella* spp., *Salmonella* spp., *Chlamydophila abortus* and *Coxiella burnetii.* Bovine viral diarrhea virus and Bovine herpes virus-1 were investigated by commercial Ag ELISA test kits (Idexx BVDV Ag/Serum Plus, Switzerland and Pulmotest BoHV-1, Belgium). See Table 1 for more details and references. The routine microbiological culture of the samples was also done under aerobic, microaerophilic and anaerobic conditions for isolation of minor infectious agents of abortion. In addition, the detection of *Neospora caninum* and *Leptospira* spp. were performed by pathological examination. The available amount of fetal heart blood was taken during postmortem examination for SAA testing. For serum separation, fetal heart blood was centrifuged at 2000 g for 5 min and separated serum samples were stored at −80 °C until use.

**Table 1:** Diagnostic protocol applied on the considered aborted fetuses.

Pathogen	Sample	Test and references
*Brucella* spp.	Abomasum content, Lung,	Bacteriological [17], PCR [18]
*Campylobacter* spp.	Abomasum content	Bacteriological [19]
*Salmonella* spp.	Liver	Bacteriological [20], PCR [21]
*Listeria* spp.	Abomasum content	Bacteriological [22]
*Chlamydophila abortus*	Liver	PCR [23]
*Coxiella burnetii*	Liver, Brain, Lung	PCR [24]
Bovine viral diarrhea virus	Spleen, Liver	Ag ELISA^a^
Bovine herpes virus-1	Spleen, Liver	Ag ELISA^b^
fungi and yeasts	Abomasum content	Microbiological [25]

^a^IDEXX BVDV Ag/Serum Plus, Switzerland. ^b^Pulmotest BoHV-1 Kit, Belgium.

### 2.2 Experimental method

Based on the diagnosis in final microbiology, pathology and molecular reports, the 46 bovine fetal abortions cases were assigned to one of two groups: Group 1: An infectious disease was diagnosed by detecting a microorganism (21 cases); Group 2: An infectious or inflammatory disease was not detected (25 cases). A commercial serum amyloid A ELISA kit was used to determine the association between fetal SAA levels in these abortion cases.

### 2.3 ELISA test for SAA

A commercial multispecies serum amyloid A enzyme linked immunosorbent assay (Multispecies SAA ELISA kit, Tridelta Development Ltd. Kildare, Ireland) was used to measure the fetal heart SAA levels for the 46 aborted bovine fetuses. Briefly, serum samples were initially diluted 1:250 in buffered saline diluent. The test kit was performed as recommended by the manufacturer. All samples were run in duplicate. The final absorbance was immediately read at 450 nm using 630 nm wavelengths in ELX 800 ELISA Microplate Spectrophotometer (Bio-Tek Instruments, Inc., USA).

### 2.4 Statistics

All statistical analyses were performed using the Mann–Whitney rank sum test. The correlation between bovine fetal heart SAA concentrations in the designated two groups was determined using Pearson correlation. *P*-value < 0.05 were considered to be statistically significant.

## 3 Results

**Figure 1:**
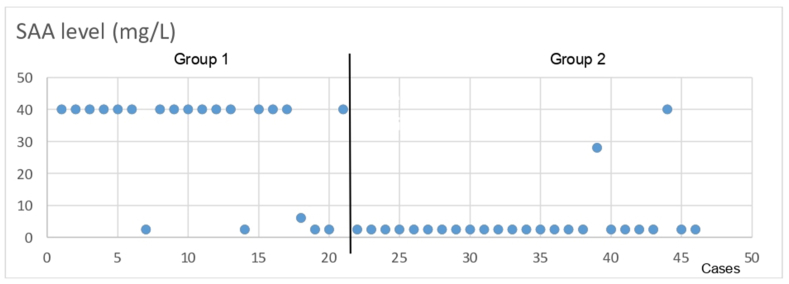
SAA concentrations in fetal heart blood in bovine abortion cases. Group 1: An infectious disease was diagnosed with an identified microorganism. Group 2: An infectious or inflammatory disease was not detected.

**Figure 2:**
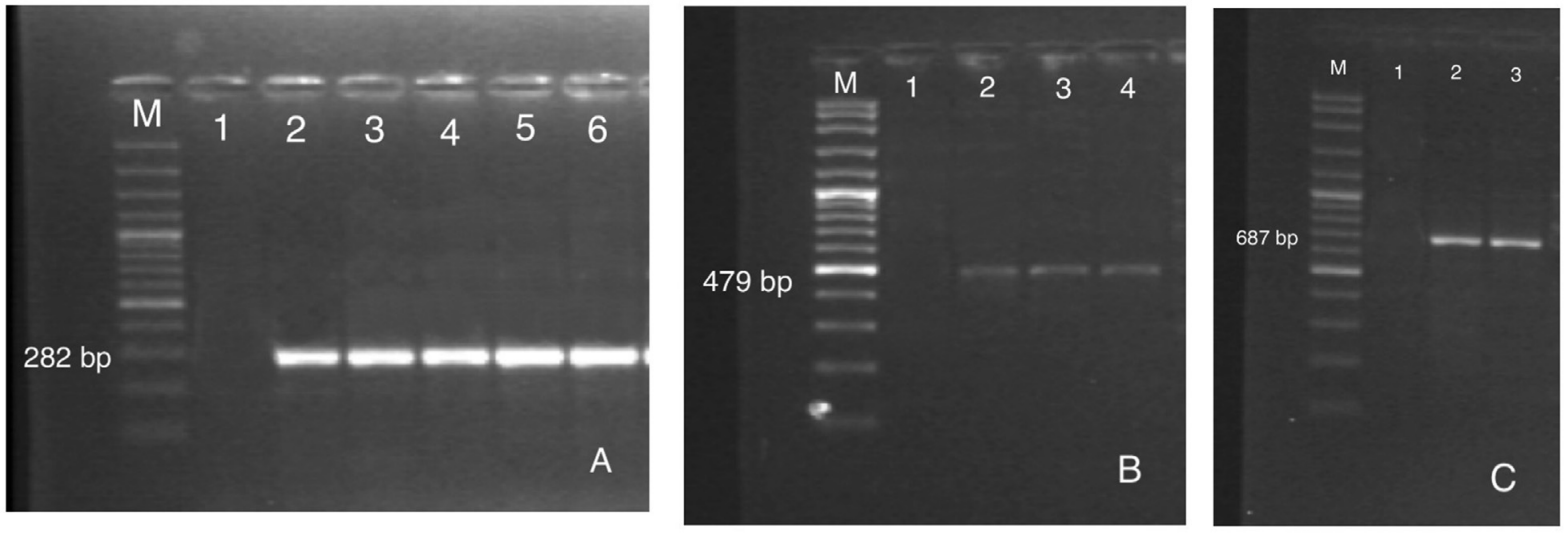
A. Agarose gel electrophoresis of *omp*2 gene PCR products of *Brucella* isolates. M: 100 bp marker (Vivantis); lane 1: negative control; lane 2: positive control (*B. melitensis* Rev-1); lanes 3–6: *Brucella* spp. positive field strains (282 bp). B. Agarose gel electrophoresis of PCR products of *C. abortus*. M: 100 bp marker (Vivantis); lane 1: negative control; lane 2: positive control DNA (*C. abortus* S26/3); lanes 2–3: *C. abortus* positive field samples (479 bp). C. Agarose gel electrophoresis of PCR products of *C. burnetii*. M: 100 bp marker (Vivantis); lane 1: negative control; lanes 2: positive control DNA; lanes 3: *C. burnetii* positive field sample (687 bp).

In group 1, SAA concentrations ranged from 6.1 to ≥40 mg/L in 17 of 21 cases in which an infectious disease agent was isolated or detected (Figure 1, Figure 2A, B, C, Table 2). These abortions were caused by bovine viral diarrhea virus (six cases), *Brucella* spp. (four cases), *Campylobacter* spp. (two cases), *Chlamydophila abortus* (two cases), *Coxiella burnetii* (one case), Bovine herpes virus-1 (one case) and yeasts (one case). Furthermore, fetal SAA concentrations were below 2.5 mg/L in the remaining four cases, in which bovine viral diarrhea virus (one case), Bovine herpes virus-1 (one case), *Campylobacter* spp. (one case), and yeasts (one case) were detected.

**Table 2:** Group 1: Bovine fetal abortion cases involving an infectious disease with a microorganism detected and their fetal SAA concentrations.

Number of cases	Diagnosis	SAA concentrations (mg/L)
7	Bovine viral diarrhea virus	≥40 mg/L (6); <2.5 mg/L (1)
4	*Brucella* spp.	≥40 mg/L (4)
3	*Campylobacter* spp.	≥40 mg/L (2); <2.5 mg/L (1)
2	*Chlamydophila abortus*	≥40 mg/L (2)
1	*Coxiella burnetii*	≥40 mg/L (1)
2	Bovine herpes virus-1	6.1 mg/L (1); <2.5 mg/L (1)
2	Yeasts	≥40 mg/L (1); <2.5 mg/L (1)

**Figure 3:**
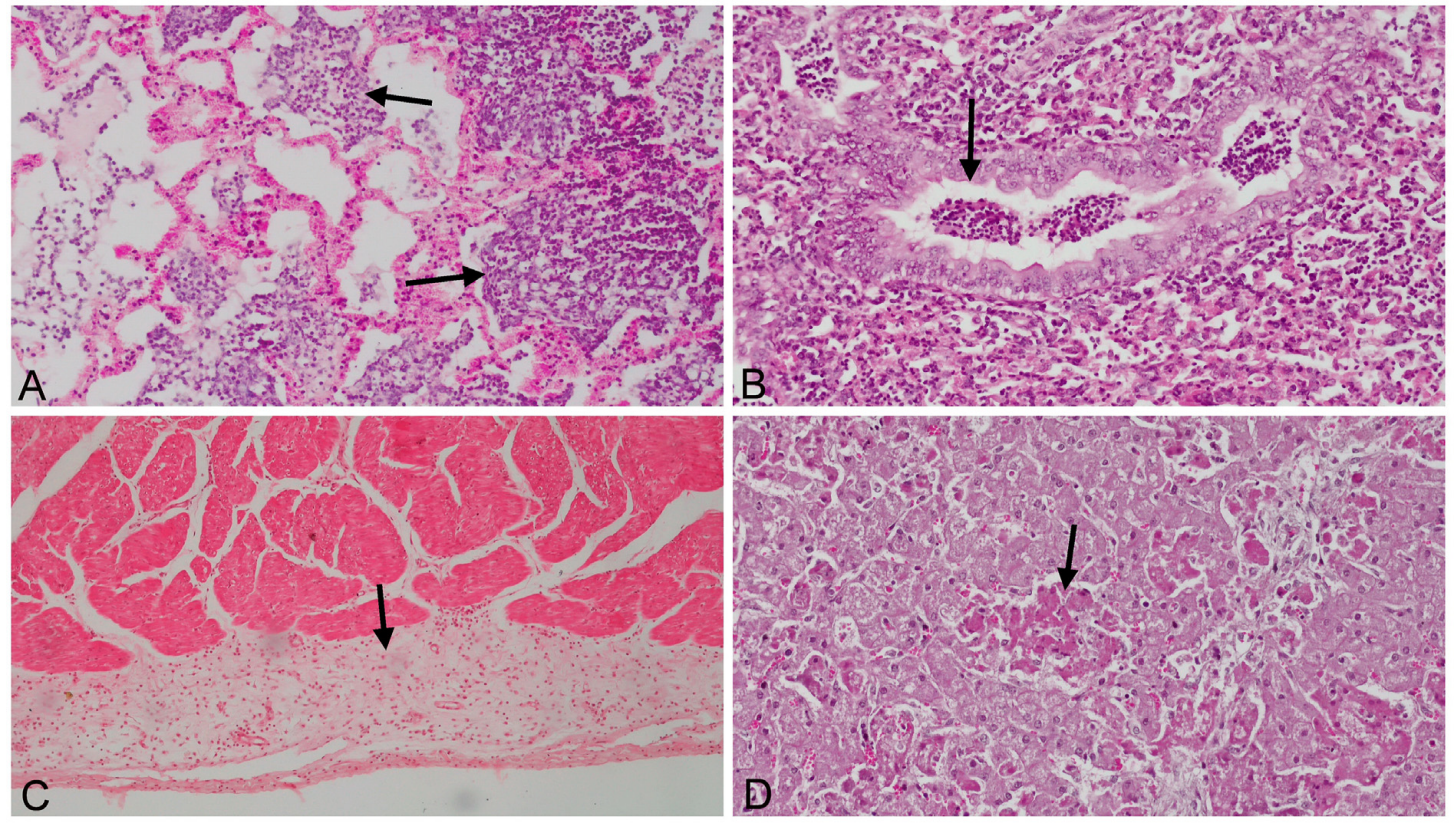
A. Fibrinous bronchopneumonia in the lungs. Neutrophil granulocytes and fibrin deposits accumulation in the alveoli (arrows). ×400 magnification. HE. B. Catarrhal bronchopneumonia in the lungs. Exudate accumulation in the bronchulus (arrow). ×400 magnification. HE. C. Fibrinopurulent pericarditis in the heart. Thickening of the pericardia due to fibrin deposits and mononuclear cell accumulation (arrow). ×400 magnification. HE. D. Multifocal necrotic hepatitis in the liver. Multifocal distributed necrotic hepatocytes (arrow). ×400 magnification. HE.

In the histopathological examination, pathological changes were present in all 21 fetuses, and included various degrees of fibrinous and catarrhal bronchopneumonia, fibrinopurulent pericarditis, and multifocal necrotic hepatitis (Figure 3A, B, C, D).

**Figure 4:**
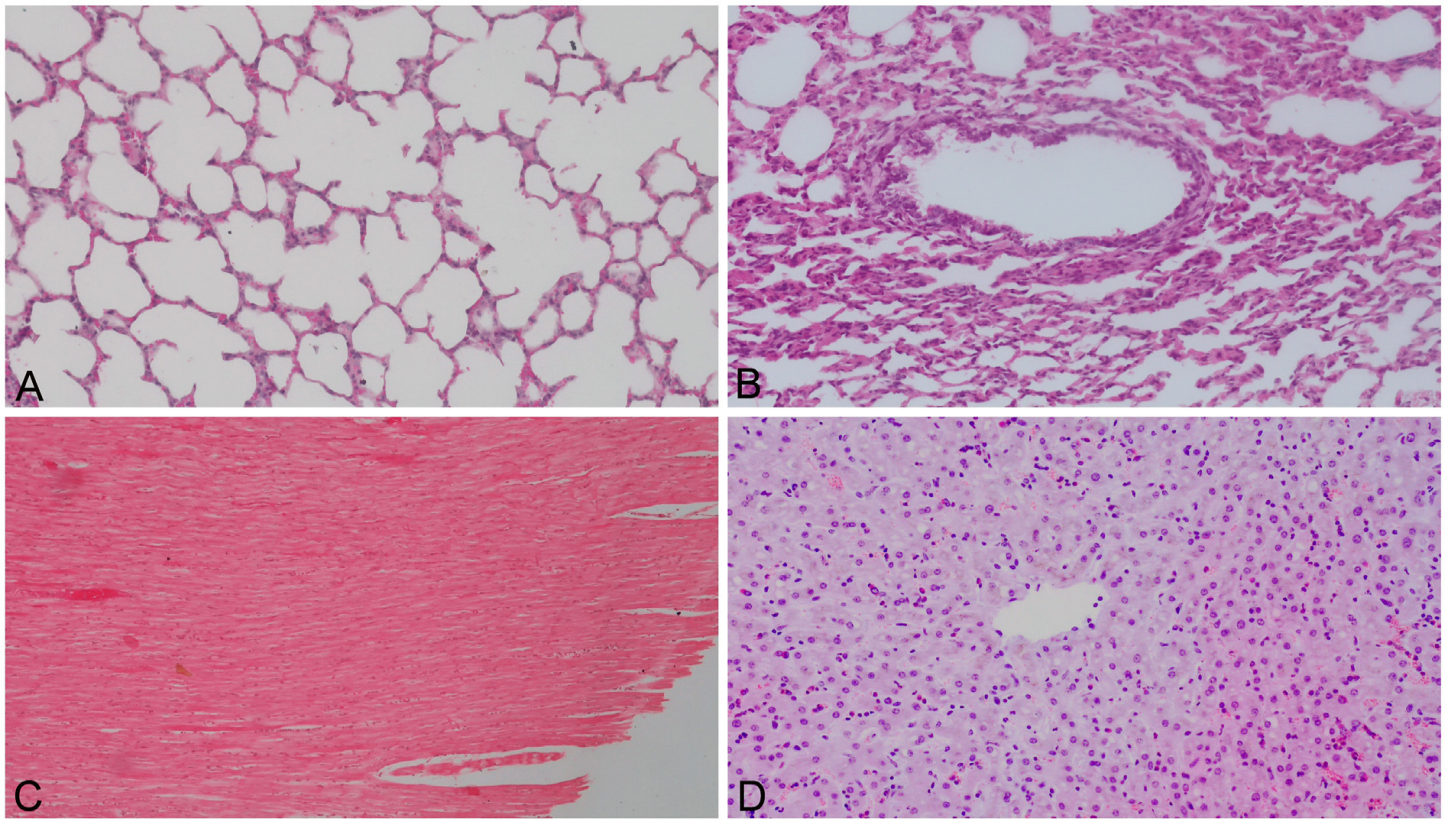
A. Lungs. Normal view of the alveoli. ×400 magnification. HE. B. Lungs. Normal view of the bronchulus. ×400 magnification. HE. C. Heart. Normal muscle filaments of the heart. ×400 magnification. HE. D. Liver. Normal appearance of the liver. ×400 magnification. HE.

In group 2, SAA levels were below 2.5 mg/L in 23 cases (Figure 1, Table 3). These abortions were due to undetermined reason (4 cases), dystocia (8 cases), hearth defect (3 cases), fetal stress (4 cases), hydrops (two cases) and umbilical cord anomaly (two cases). In the remaining two cases which included dystocia (one case) and fetal stress (one case), SAA concentration was detected as 28 and ≥40 mg/L, respectively. In the microscopic evaluation, there were no gross and histopathological changes in 4 abortion cases of undetermined reason. Additionally, in the 25 fetuses, no inflammatory or necrotic changes observed on pathological examination (Figure 4A, B, C, D).

**Table 3:** Group 2: Bovine fetal abortion cases without infectious disease or inflammation and their fetal SAA concentrations.

Number of cases	Diagnosis	SAA concentrations (mg/L)
4	Undetermined abortion	<2.5 mg/L (4 cases)
9	Dystocia	<2.5 mg/L (8 cases); 28 mg/L (1 case)
3	Hearth defect	<2.5 mg/L (3 cases)
5	Fetal stress	<2.5 mg/L (4 cases); ≥40 mg/L (1 case)
2	Hydrops	<2.5 mg/L (2 cases)
2	Umbilical cord anomaly	<2.5 mg/L (2 cases)

Additionally, a statistically significant difference was found between group 1 and group 2 with regard to SAA levels (P ≤ 0.001).

## 4 Discussion

SAA levels have been investigated as biomarkers of disease in human and domestic animals in recent years [10, 12, 13, 14, 15]. In the present study, the fetal heart blood SAA concentrations in aborted bovine fetuses were evaluated and the relationship between the level of SAA and infections was investigated. To the best of our knowledge, this is the first report addressing the relationship between infectious abortions and fetal heart blood SAA levels in bovines.

The results of the present work demonstrated that the fetal heart blood SAA concentrations were significantly elevated from 6.1 to ≥40 mg/L in 17 of 21 cases where an infectious disease was diagnosed from aborted bovine fetuses. The high SAA levels were detected in six of seven bovine viral diarrhea disease cases, four brucellosis cases, two of three campylobacteriosis cases, two *Chlamydophila abortus* infection cases, one of two infectious bovine rhinotracheitis cases, one of two yeast infection cases, and one Q fever case. Erol et al. [10] investigated the fetal heart blood SAA concentration in cases of aborted equine fetuses and found elevated levels 4.8 to ≥40 mg/L in 17 of 23 cases where an infectious agent was identified in tissues. Jawor et al. [14] compared SAA concentrations in blood plasma samples of cases of bovine perinatal mortality due to infection *in utero* or unexplained cases. They reported that concentrations of SAA in the stillborn calves with bacterial infection were significantly higher than those in the group of unexplained deaths. These findings are consistent with the results of our research. On the other hand, in our study, fetal SAA concentrations were less than 2.5 mg/L in four cases where an infectious disease was diagnosed as bovine viral diarrhea disease, campylobacteriosis, infectious bovine rhinotracheitis, and yeast infection. In these four cases, the time interval between abortion and admission of the fetus to the laboratory was 2–8 h. However, there was no information on how long the fetuses remained dead in the uterus. The low level of fetal SAA in these four cases could be due to the long time the dead fetuses remained in the uterus before abortion. The effect of environmental temperature and time on fetal SAA concentration was investigated in another study in which it was reported that fetal SAA concentration significantly reduced after 72 h of incubation at 37 °C [10]. This report may also explain the values of these four cases with the low concentration of fetal SAA that might have died *in utero* at least 3 day before abortion.

In our study, serum amyloid A concentrations were found to be below 2.5 mg/L in 23 of 25 cases from group 2. However, two of the 25 cases in group 2 had 28 and ≥40 mg/L fetal SAA levels. One of the two cases had a fetal stress and the other was diagnosed with dystocia. The reason for high SAA concentrations in these cases may be unknown infectious or chemical agents. Similar results to ours were reported by Erol et al. [10] and Jawor et al. [13] in groups where microorganisms could not be isolated. These results show that fetal blood SAA concentrations are low in non-infectious bovine abortion cases.

There is an increasing trend in the use of SAA in determination of diagnosis, prognosis, pathophysiology and immunology in animals [10, 12, 13, 14, 15]. Serum amyloid A concentrations are generally low after birth of calves without infection [14]. The infected fetuses can produce large molecules such as pathogen-specific immunoglobulins and serum amyloid A [26, 27]. The increased concentrations of SAA in bovine fetal heart blood and its association with infectious disease is significant evidence. SAA levels could be used especially for further investigation of abortion cases in which a definitive diagnosis has not been made [10].

## 5 Conclusion

The present findings suggest that SAA levels in fetal heart blood from bovine fetuses is potentially a novel marker for distinguishing between infectious and non-infectious bovine abortion cases. Fetal SAA is easily quantifiable and highly sensitive and has potential for diagnosis of infectious bovine abort cases.

## Declarations

### Author contribution statement

Zeki Aras: Conceived and designed the experiments; Performed the experiments; Contributed reagents, materials, analysis tools or data; Wrote the paper.

Orhan Yavuz: Performed the experiments; Contributed reagents, materials, analysis tools or data.

### Funding statement

This research did not receive any specific grant from funding agencies in the public, commercial, or not-for-profit sectors.

### Data availability statement

No data was used for the research described in the article.

### Declaration of interest’s statement

The authors declare no conflict of interest.

### Additional information

No additional information is available for this paper.

## References

[1] Wathes D.C., Oguejiofor C.F., Thomas C., Cheng Z. (2020). Importance of viral disease in dairy cow fertility. Engineering.

[2] Carpenter T.E., Chrièl M., Andersen M.M., Wulfson L., Jensen A.M., Houe H., Greiner M. (2006). An epidemiologic study of lateterm abortions in dairy cattle in Denmark, July 2000–August 2003. Prev. Vet. Med..

[3] Kafi M., Zibaei M., Rahbari A. (2007). Accuracy of oestrus detection in cows and its economic impact on Shiraz dairy farms. Iran. J. Vet. Res..

[4] Noaman V., Nabinejad A.R. (2020). Seroprevalence and risk factors assessment of the three main infectious agents associated with abortion in dairy cattle in Isfahan province, Iran. Trop. Anim. Health Prod..

[5] Cray C., Zaias J., Altman N.H. (2009). Acute phase response in animals: a review. Comp. Med..

[6] Knebel F.H., Albuquerque R.C., Massaro R.R., Maria-Engler S.S., Campa A. (2013). Dual efect of serum amyloid A on the invasiveness of glioma cells. Mediat. Inflamm..

[7] Sandri S., Urban B.A., Fernandes I., de Oliveira E.M., Knebel F.H., Ruano R., Zugaib M., Filippin-Monteiro F., Bevilacqua E. (2014). Serum amyloid A in the placenta and its role in trophoblast invasion. PLoS One.

[8] Kovacevic A., Hammer A., Sundl M., Pfister B., Hrzenjak A., Ray A., Ray B.K., Sattler W., Malle E. (2006). Expression of serum amyloid A transcripts in human trophoblast and fetal- derived trophoblast-like choriocarcinoma cells. FEBS Le.

[9] Borbely A.U., Sandri S., Fernandes I.R., Prado K.M., Cardoso E.C., Correa-Silva S., Albuquerque R., Knöfler M., Beltrão-Braga P., Campa A., Bevilacqua E. (2014). The term basal plate of the human placenta as a source of func onal extravillous trophoblast cells. Reprod. Biol. Endocrinol..

[10] Erol E., Jackson C., Horohov D., Locke S., Smith J., Carter C. (2016). Elevated serum amyloid A levels in cases of aborted equine fetuses due to fetal and placental infections. Theriogenology.

[11] Castellano M., Silva-Álvarez V., Aversa-Marnai M., Lamas-Bervejillo M., Quartiani I., Perretta A., Villarino A., Ferreira A.M. (2020). Serum amyloid A is a positive acute phase protein in Russian sturgeon challenged with Aeromonas hydrophila. Sci. Rep..

[12] Sharifiyazdia H., Nazifi S., Nikseresht K., Shahriari R. (2012). Evaluation of serum amyloid A and haptoglobin in dairy cows naturally infected with brucellosis. J. Bacteriol. Parasitol..

[13] Jawor P., Stefaniak T., Mee J.F. (2017). Immune and inflammatory biomarkers in cases of bovine perinatal mortality with and without infection in utero. J. Dairy Sci..

[14] Jawor P., Mee J.F., Stefaniak T. (2018). Perinatal immuno/inflammatory responses in the presence or absence of bovine fetal infection. BMC Vet. Res..

[15] Joshi V., Gupta V.K., Bhanuprakash A.G., Mandal R.S.K., Dimri U., Ajith Y. (2018). Haptoglobin and serum amyloid A as putative biomarker candidates of naturally occurring bovine respiratory disease in dairy calves. Microb. Pathog..

[16] Presnell J.K., Schreibman M.P. (1977).

[17] Alton G.G., Jones L.M., Angus R.D., Verger J.M. (1988).

[18] Cloeckaert A., Verger J.M., Grayon M., Grepinet O. (1995). Restriction site polymorphism of the genes encoding the major 25 kDa and 36 kDa outer-membrane proteins of *Brucella*. Microbiology.

[19] World Organization for Animal Health (OIE) (2017). Manual of Diagnostic Tests and Vaccines for Terrestrial Animals, 3.4.4. http://www.oie.int/fileadmin/Home/eng/Health_standards/tahm/3.04.04_BGC.pdf.

[20] World Organization for Animal Health (OIE) (2016). Manual of Diagnostic Tests and Vaccines for Terrestrial Animals, 3.9.8. http://www.oie.int/fileadmin/Home/eng/Health_standards/tahm/3.09.08_SALMONELLOSIS.pdf.

[21] Beuzon C.R., Schiaffino A., Leori G., Cappuccinelli P., Rubino S., Casadesus J. (1997). Identification of *Salmonella abortusovis* by PCR amplification of a serovar-specific IS*200* element. Appl. Environ. Microbiol..

[22] Eld K., Danielsson-Tham M.L., Gunnarsson A., Tham W. (1993). Comparison of a cold enrichment method and the IDF method for isolation of *Listeria monocytogenes* from animal autopsy material. Vet. Microbiol..

[23] Crealan J., Mc Cullough L. (2000). Evaluation of strain specific primer sequnces from an abortifacient strain of ovine *Chlamydophila abortus (Chlamydia psittaci)* for the detection of EAE by PCR. FEMS Microbiol. Lett..

[24] Hoover T.A., Vodkin M.H., Williams J.C. (1992). A *Coxiella burnetii* repeated DNA element resembling a bacterial insertion sequence. J. Bacteriol..

[25] Kirkbride C.A. (1976). Mycotic abortion. Theriogenology.

[26] Erol E., Jackson C.B., Steinman M., Meares K., Donahoe J., Kelly N., Locke S., Smith J.L., Carter C.N. (2015). A diagnostic evaluation of real-time PCR, fluorescent antibody and microscopic agglutination tests in cases of equine leptospiral abortion. Equine Vet. J..

[27] Wilson T.C., Bachurski C.J., Ikegami M., Jobe A.H., Kallapur S.G. (2005). Pulmonary and systemic induction of SAA3 after ventilation and endotoxin in preterm lambs. Pediatr. Res..

